# Purple Chromoprotein Gene Serves as a New Selection Marker for Transgenesis of the Microalga *Nannochloropsis oculata*

**DOI:** 10.1371/journal.pone.0120780

**Published:** 2015-03-20

**Authors:** Chen-Han Shih, Hsiao-Yin Chen, Hung-Chieh Lee, Huai-Jen Tsai

**Affiliations:** 1 Institute of Molecular and Cellular Biology, National Taiwan University, Taipei, Taiwan; 2 Institute of Biomedical Sciences, MacKay Medical College, New Taipei City, Taiwan; IPK, GERMANY

## Abstract

Among the methods used to screen transgenic microalgae, antibiotics selection has raised environmental and food safety concerns, while the observation of fluorescence proteins could be influenced by the endogenous fluorescence of host chloroplasts. As an alternative, this study isolated the purple chromoprotein (CP) from *Stichodacyla haddoni* (shCP). A plasmid in which shCP cDNA is driven by a heat-inducible promoter was linearized and electroporated into 2.5×10^8^ protoplasts of *Nannochloropsis oculata*. Following regeneration and cultivation on an f/2 medium plate for two weeks, we observed 26 colonies that displayed a slightly dark green coloration. After individually subculturing and performing five hours of heat shock at 42°C, a dark brown color was mosaically displayed in five of these colonies, indicating that both untransformed and transformed cells were mixed together in each colony. To obtain a uniform expression of shCP throughout the whole colony, we continuously isolated each transformed cell that exhibited brown coloration and subcultured it on a fresh plate, resulting in the generation of five transgenic lines of *N*. *oculata* which stably harbored the shCP gene for at least 22 months, as confirmed by PCR detection and observation by the naked eye. As shown by Western blot, exogenous shCP protein was expressed in these transgenic microalgae. Since shCP protein is biodegradable and originates from a marine organism, both environmental and food safety concerns have been eliminated, making this novel shCP reporter gene a simple, but effective and ecologically safe, marker for screening and isolating transgenic microalgae.

## Introduction

The genetic modification of algae potentially offers an important approach to improve aqua- and agricultural applications, food quality and human health [1]. Generally, all transformation protocols for generating transgenic organisms require marker genes to identify and isolate transformed algae and may be classified as follows: (*i*) auxotrophic markers, such as *Nit1* [2] and *Arg7* [3], which require a corresponding normal gene to rescue the microalgae mutants and keep them alive on conditional medium; (*ii*) selection marker genes, such as *CRY1*, *Ble*, *NptII* and *AphVIII* [4–7], which are commonly used to select microalgal transformed cells by conferring genes resistant to antibiotics or herbicides and allowing, in turn, host cells to survive in medium containing antibiotics or herbicides; and (*iii*) reporter genes, which do not exert selective pressure on transformed cells [8], such as genes encoding fluorescence proteins [9, 10] or bioluminescent proteins [11].

Most selective marker genes have some disadvantages. In the case of auxotrophic selection markers, it is argued that auxotrophic mutant strains are required, making this marker suitable for only haploid genome algae [12]. With fluorescent proteins, the large distribution of chlorophyll and pigments in host microalgae might mask or otherwise impede the observation of fluorescent signals, resulting in poor identification of the transformed cells under fluorescence microscopy. Additionally, fluorescent instrumentation is a requirement for observation of these markers. Antibiotic and herbicide selection markers are restricted because many algae have natural resistance to most antibiotics or herbicides [13]. Moreover, many marine algae grow in highly saline conditions, which reduce the activity of antibiotics [14]. In fact, marker genes applied in genetically modified organisms (GMO) have raised public concern over environmental and food safety. The European Parliament and Council of the European Union have declared that release of transgenic organisms into the environment is conditional on the removal of antibiotics-resistant genes for biomedical and/or agricultural applications in view of potential adverse effects on human health and the environment. As a result, biosafety seems to preclude the use of antibiotics- and herbicides-resistant genes as marker genes for screening transgenic algae. Over the years, a variety of marker excision methods have been developed [15–17]. For example, direct-repeat-mediated excision via homologous recombination [18], co-transformation [19] and site-specific recombination (SSR), such as *Cre*/*lox* [20], *FLP*/*FRT* [21] and *R*/*RS* systems [22], have been developed to produce marker-free transgenic algae and other plants [23–26]. However, some drawbacks have been reported that limit the application of these marker excision methods. For example, transformation lines generated from site-specific recombination (SSR) are genetically unstable, making it difficult to control the production of marker-free transplastomic clones [27, 28]. SSR is applicable only to sexually propagated plants, and it is a time-consuming process [16, 26, 29]. Moreover, the *Cre*/*lox* recombination system requires retransformation treatment which is both labor-intensive and time-consuming [20, 30], and the expression of recombinase genes for prolonged periods in plant cells might cause abnormalities in transgenic plants [16, 29, 31]. Finally, Kilian et al. [32] knocked out the endogenous nitrate or nitrite reductase gene by inserting the desired transgene, allowing transgenic cells to be screened on nitrite-containing medium. Although this approach solves some disadvantages of using antibiotics- and herbicides-resistant genes, developing an alternative selection marker that is a simple, rapid and ecologically safe is still necessary.

We previously isolated a cDNA fragment encoding a novel GFP-like chromoprotein from the carpet anemone *Stichodactyla haddoni*, termed shCP [33]. The shCP protein consists of 227 amino acid residues, sharing 96% identity with the GFP-like chromoprotein of *Hecteractis crispa*. This protein possesses a maximum absorption wavelength peak (λ_max_) at 574 nm, resulting in a purple color. Microalgae are photosynthetic microorganisms, and some species have been utilized in aquaculture as essential nutritional feed for the production of farmed organisms and other commercially important aquaculture species [34]. *Nannochloropsis oculata* is the most frequently used microalga in aquaculture applications [35–38]. *N*. *oculata* is an ovoid-shaped marine unicellular microalga that grows in a wide range of conditions that vary by pH, temperature and salinity. It also possesses a high rate of biomass production and a high content of unsaturated fatty acids [35], which are associated with health benefits [39]. Furthermore, *N*. *oculata* becomes a potentially attractive bioreactor for biofuel production and live feed for fish and shellfish larvae [40]. In this study, we demonstrated that this cDNA fragment encoding the purple chromoprotein shCP is an effective and an ecologically friendly selection marker gene for the transformed *N*. *oculata*, enabling the identification and isolation of transgenic microalgae by direct visual detection without the necessity of either auxotrophic mutants or complicated fluorescence equipment.

## Results

### Screening of stable transformants of *N*. *oculata* using purple chromoprotein as a selective marker

**Fig 1:**
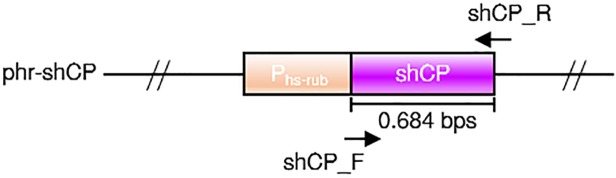
Plasmid constructs used to generate the transgenic lines of *N*. *oculata*. Plasmid phr-shCP, in which the coding region of the purple chromoprotein (CP) cDNA (purple bar) of *Stichodactyla haddoni* (shCP) is driven by inducible heat-shock promoter and Rubisco promoter (P_hs-rub_). Primer sets used to determine the existence of transferred gene in microalgae cells are shown.

**Fig 2:**
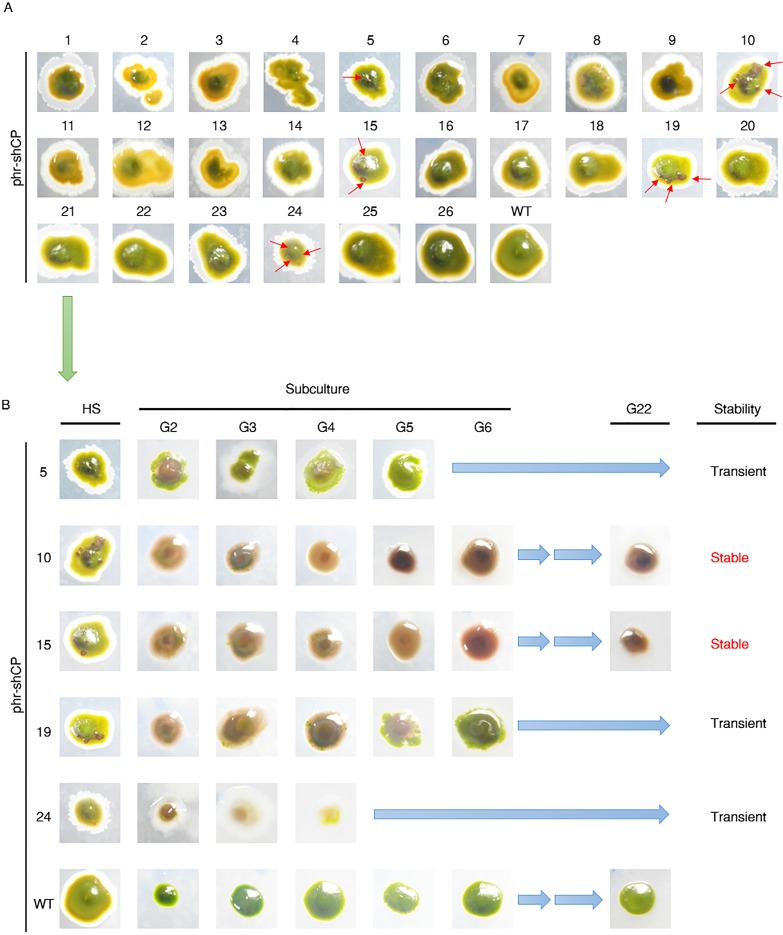
Using purple chromoprotein to screen and isolate the transformed colonies containing the exogenous shCP gene. (A) After electroporation with plasmid phr-shCP, 26 colonies of *N*. *oculata* were grown on f/2 medium plates for two weeks, followed by heat shock at 42°C for 5 hr. Dark brown color presented by reporter chromoprotein was observed in some cells within each colony, as indicated by the red arrow. The wild-type colony served as a negative control. (B) The putative transformants of *N*. *oculata* were purified and subcultured to the second round of replating (G2), the third round of replating (G3) and so on during each of the next 22 months. In contrast to the green color presented in the wild-type strain, a dark brown color was exhibited in the whole colony of transgenic strains, especially in transgenic lines CP10 and CP15. “HS” indicates that heat shock treatment was performed. “Transient” indicates that the foreign shCP gene could only be temporarily transformed and expressed in a few rounds of replating. “Stable” indicates that foreign protein could be continuously expressed beyond the 21^st^ round of replating.

As depicted in Fig. 1, the expression plasmid we constructed and used in this study contains a shCP cDNA driven by *HSP70A*::*RBCS2* promoter that can be induced by light and heat shock [41, 42]. This construct was linearized and electroporated into wild-type *N*. *oculata* protoplast cells. After cultivation of all treated algal cells (2.5 × 10^8^ cells) for two weeks on an f/2 medium plate, 94 colonies were grown (S1A Fig.). Many colonies exhibited a green color, but we observed 26 colonies that displayed a slightly dark green coloration (S1B Fig.). Each one of these 26 colonies was individually subcultured and selected for further heat-shock treatment. Finally, after 5 hours of heat shock at 42°C, a dark brown color was observed in five of these transformants, which were named CP5, CP10, CP15, CP19 and CP23 (Fig. 2A and 2B). However, instead of a uniform expression of shCP throughout the whole colony, we noticed only a dark brown color mosaically displayed in each colony, indicating that both untransformed and transformed cells were mixed together in each colony. Therefore, to avoid the contamination of untransformed wild-type cells on the f/2 medium plate, we directly and continuously isolated each transformed cell and subcultured it on a fresh plate. As a result, the five strains of phr-shCP-transferred microalgae noted above were successfully maintained as independent transgenic lines that displayed a uniformly dark brown color throughout the whole colony (Fig. 2B). After continuous subculture with several rounds of replating, we generated two pure transgenic lines, CP10 and CP15, which could express the shCP gene up to at least the twenty-first round of replating (Fig. 2B; Table 1), indicating that the foreign shCP gene could be stably transformed in these two transgenic lines for at least 22 months. In contrast, three transformants, including CP5, CP19 and CP23, failed to continuously express the shCP gene since those transformed cells eventually turned green, similar to the wild-type microalgae, when they were subcultured up to the second, sixth and fourth rounds of replating, respectively (Fig. 2B; Table 1), indicating that the exogenous DNA in these lines might have been transient during subculture.

**Table 1:** The long-term stability of transgenic lines harboring the exogenous phr-shCP DNA fragment in *N. oculata*.

Plasmid^a^	Transgenic line	Rounds of replating ^b^	Cultivation period^c^	Stability ^d^
phr-shCP	CP5	21	22 months	Transient (until the 2^nd^ r.o.r.)
	CP10	21	22 months	Stable
	CP15	21	22 months	Stable
	CP19	21	22 months	Transient (until the 6^th^ r.o.r.)
	CP23	21	22 months	Transient (until the 4^th^ r.o.r.)

^a^ Genetic transformation with linearized plasmid phr-shCP.

^b^ r.o.r.: Rounds of replating indicate the number of months algal colonies were subcultured after initial selection.

^c^ The total incubation period after transformation.

^d^ The existence of exogenous phr-shCP in the transformants exhibiting a dark brown coloration after heat-shock treatment.

In order to confirm the reproducibility of this technology, we repeated the transformation experiment following the same conditions as described above. After electroporation, algal cells (2.5 × 10^8^ cells) were cultured on an f/2 medium plate for two weeks. A total of 384 colonies were grown on the plate (S2A Fig.). All but 15 colonies displayed a green coloration. These 15 colonies, which displayed a slightly dark green color, were individually picked up and subcultured on a fresh f/2 medium plate for two weeks (S2B Fig.). When they were cultured for one more week after heat-shock treatment, a dark brown coloration was displayed in ten transformants, termed CP^2nd^ 1, CP^2nd^ 4∼5 and CP^2nd^7∼14. Similar to the first experiment, the dark brown color was also mosaically displayed in each colony (S3A Fig.). However, after these ten colonies were continuously isolated and subcultured, a dark brown coloration was presented uniformly throughout the whole colony (S3B Fig.). Similar to transgenic lines CP10 and CP15 in the first experiment, these pure phr-shCP-transferred microalgal varieties were able to express the shCP gene up to the 15^th^ round of replating (S3B Fig.). The results obtained from these two independent experiments were summarized in S1 Table. This line of evidence suggested that the cDNA fragment encoding the purple chromoprotein shCP is a useful and convenient marker gene that allows differentiation between the pure and stable transformed clones, as indicated by the dark brown coloration, and the contaminated and transient transformed colonies, as indicated by partial dark brown or green coloration, using simple visual detection.

### PCR detection of the transferred shCP gene

**Fig 3:**
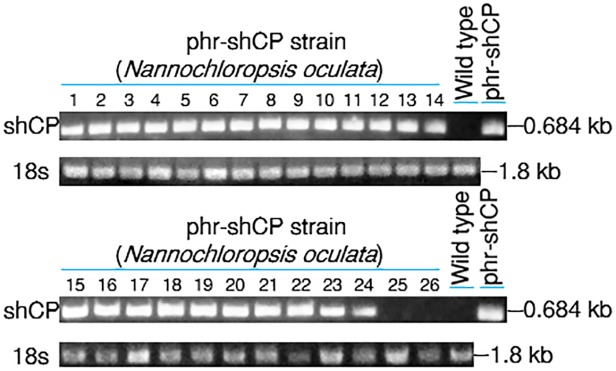
Using PCR to detect of the existence of transferred gene in the transformed *N*. *oculata*. Plasmid phr-shCP was used to transform *N*. *oculata*. After transformation, all microalgae cells were cultured, and the putative clones were selected to determine the existence of shCP fragment in *N*. *oculata* transformed with phr-shCP at the expected size of 0.684 kb. The wild-type (WT) strain served as a negative control, whereas plasmid phr-shCP served as a positive control. The small subunit 18S ribosomal RNA (18s) served as the internal control.

To confirm whether the exogenous shCP cDNA exists in host cells, genomic DNAs were isolated from the 26 subcultured strains of *N*. *oculata* which were grown on plates after gene transfer of phr-shCP. PCR detection showed that no DNA fragment had been amplified from the wild-type colony. On the other hand, out of 26 colonies, a 684-bp fragment was amplified from 24, with only CP25 and CP26 failing to yield PCR products (Fig. 3). However, as mentioned above, only five transformants, including CP5, CP10, CP15, CP19 and CP23, presented dark brown coloration, indicating that the exogenous shCP gene in some transformants might have been silenced, resulting in the absence of dark brown coloration, even though the shCP gene had been transferred. Based on the number of transformants expressing shCP, we calculated the expression rate of foreign shCP gene in this study to be around 20%, or five out of 24 PCR-positive colonies.

Interestingly, when we used PCR to detect the genomic DNAs extracted from these five lines after they had been subcultured for 15 months, we found that a 684-bp DNA fragment was only amplified in the transformed cells from lines CP10 and CP15 whose dark brown coloration remained unchanged. However, after subculture for 15 months, no amplified DNA was detected in the transformed cells from lines CP5, CP19 and CP23 whose dark brown color had reverted to green by the loss of the shCP gene. Therefore, we concluded that the dark brown color appearing in the transformed cells from lines CP10 and CP15 resulted from the presence of the shCP protein encoded from the exogenous shCP gene, whereas the green color appearing in the transformed cells from lines CP5, CP19 and CP23 resulted from the absence of the shCP protein for an unknown reason. In the second transformation experiment detailed above, the 15 colonies that displayed a slightly dark green coloration after subculture tested positive for the shCP gene by PCR. PCR detection was also positive for the colonies from ten stable lines, CP^2nd^ 1, CP^2nd^ 4∼5 and CP^2nd^7∼14, when they were at the 5^th^ round of replating (S4 Fig.).

### Dark brown coloration in the transformed cells

**Fig 4:**
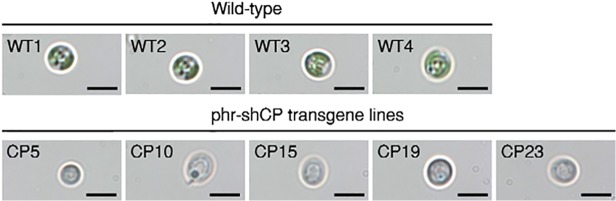
Visualization of marker gene in the transformed strain by stereo investigator and neurolucida microscopy image. Visualization of transgenic cells (CP) and wild-type cells (WT) of microalgae by bright-field microscopy at higher (2,000 X) magnification. The shCP protein was expressed in transgenic lines containing plasmid phr-shCP. (Left column): purple chromoprotein that presented in the cytoplasm of transgenic cells was mixed with endogenous intercellular chlorophyll, resulting in dark brown color; (WT1-WT4): green color that presented in nontransgenic wild-type cells served as a negative control. Scale bars: 5 μm.

To analyze the distribution of exogenous shCP protein in the transformants of *N*. *oculata*, we employed Stereo Investigator and Neurolucida Microscopy to observe the expression of exogenous shCP gene in the transformants. For comparison and confirmation of bright-field images, we observed a single cell of transformed algal strain and wild-type strain at higher magnification. Under this field magnified 2000 times, the image clearly revealed the difference of phenotype between the transformed cells and wild-type cells. The wild-type cells were green and displayed a round shape with diameter of 2–4 μm (Fig. 4, WT1-WT4). Similar to the wild type, the shape and size of transgenic cells remained unchanged (Fig. 4, Left column), but unlike the wild type, the dark brown coloration was clearly observed in the cells of transgenic strains harboring phr-shCP (Fig. 4, Left column). The appearance of exogenous purple chromoprotein encoded by the transferred shCP cDNA in the transformants overlapped with endogenous green chlorophyll and other antenna colors, causing a change in color to dark brown. We also observed that the exogenous shCP protein had largely accumulated in the cytosol. In contrast, the cells of the wild-type strain had no exogenous shCP gene and presented, instead, their original green coloration (Fig. 4, WT1-WT4). The color change in the transformed strains could be easily observed not only in cells, but also in colonies (Fig. 2), by the naked eye.

### Western blot analysis of exogenous shCP protein in the transformed strains

**Fig 5:**
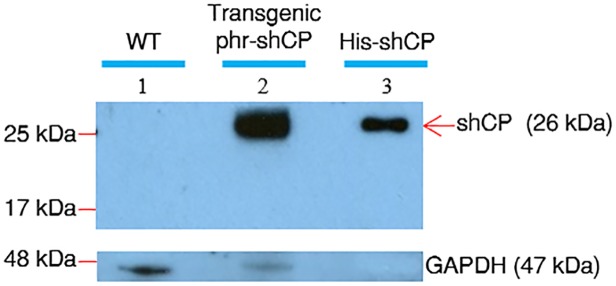
Detection of shCP protein encoded from plasmid phr-shCP in the transgenic line of *N*. *oculata*. The polyclonal antibody against shCP was used to perform immunoblot analysis of total soluble proteins extracted from wild-type (WT) algal cells (60 μg), as well as transgenic algal cells of line CP10 (50 μg; transgenic phr-shCP). The recombinant protein shCP (His-tagged shCP; His-shCP) extracted from *E*. *coli* served as a positive control. The His-tagged shCP produced by *E*. *coli* and the non-His-tagged shCP produced by *N*. *oculata* were detected, as indicated by the arrow. Antibodies against glyceraldehyde 3-phosphate dehydrogenase (GAPDH) served as an internal control.

To confirm expression of the recombinant shCP protein in the transgenic microalga, we extracted total soluble proteins from transgenic line CP10 and analyzed them by Western blot using polyclonal antibody against shCP protein. Similar to the recombinant shCP produced by *E*. *coli*, which served as a positive control (Fig. 5, lane 3), a positive hybridization band with approximate molecular weight of 26 kDa was detected in the proteins extracted from the algal cells of transgenic strain CP10 (Fig. 5, lane 2). However, no signal recognized by antibody against shCP was shown for the proteins extracted from wild-type algal cells (Fig. 5, lane 1). Even though total protein (60 μg) load on gel from wild-type exceeded that from transgenic strain CP10 (50 μg), as indicated by antibody against glyceraldehyde 3-phosphate dehydrogenase (GAPDH) used as internal control, no detectable band was shown on the gel. This line of evidence suggested that the heterologous shCP protein was correctly expressed by transformed algal cells derived from the transgenic line CP10 of *N*. *oculata*.

## Discussion

We are the first to use the purple chromoprotein of sea anemone as a selection marker in microalgae transformation. This approach presents a viable alternative to the use of drug-resistant genes in plant transgenesis, including microalgae. In this study, based on phenotypic coloration, genetic analysis, and Western blot analysis, we proved that the purple chromoprotein gene can be directly expressed in *N*. *oculata*. This selection marker was, moreover, successfully used to isolate two transgenic lines stably harboring the shCP gene by the twenty-first round of replating. More importantly, we could identify the transformed cells directly and simply by visual observation, thereby helping researchers to screen stably transformed algal varieties.

### Using chromoprotein gene for screening transgenic microalgae

Kilian et al. [32] demonstrated the use of homologous recombination to substitute the endogenous gene encoding nitrate or nitrite reductase for the desired transgene, allowing transgenic cells to be screened on nitrite-containing medium. This selection strategy for screening transgenic algae is more advantageous than using such conventional approaches as antibiotic and herbicide markers, auxotrophic markers or reporters. However this screening method still requires the implementation of tedious procedures. For example, each colony must be transferred to the nitrite-only medium, and the endogenous gene must be destroyed. In this study, we used the purple chromoprotein shCP isolated from *S*. *haddoni* as an excellent alternative method of screening for the transgenic oil-producing alga *Nannochloropsis* sp. The results can be readily visualized by the naked eye without the need for a special medium to maintain selective pressure or the need for special equipment to observe the fluorescent reporter. Moreover, endogenous genes of transformants are left intact without being inserted by the foreign DNA fragment.

Compared to the efficiency observed with transformed intact *N*. *gaditana* (12.5×10^–6^) and *Nannochloropsis sp*. (∼2500 transformants/μg DNA [32, 43]) the transformation efficiency of this study is lower. Since we used pepsin at a working pH of 2.0 to remove the cell wall of *N*. *oculata*, the cells without cell wall protection could be easily damaged or lethal by environmental cue, including physical shearing, osmosis and pH [44–46]. Therefore, out of 2.5×10^8^ cells, only a few hundred cells are ultimately capable of growing on plates after a two-week culture. Nonetheless, a hundred putative cells provide a sufficient number for screening out the transformed cells. Otherwise, screening would be complicated by the high numbers of surviving cells able to grow on nonselective plates. In our case, for each transformation of *N*. *oculata* protoplasts, more than 10 transgenic colonies were obtained after cultivation. Therefore, these numbers, along with the reduction in false-positive clones, make this process easy to apply for any reasonably equipped lab without any extensive labor or screening requirements.

### Low possibility of false-positive occurrence using shCP selection marker gene

Chen and Melis [47] reported the occurrence of false-positive clones after transformation by the high frequency of rRNA mutation in cases where antibiotics-resistant genes were used as selection markers to screen algae during transformation. This method reduces selection efficiency and increases the cost and labor involved in discarding nontransformants. Moreover, Day and Goldschmidt-Clermont [17] reported on the use of antibiotics- or herbicide-resistant genes as selection markers. They found that the antibiotics or herbicide in the medium surrounding transformants could become less concentrated, thereby affording protection to neighboring nontransformed plastids and cells and, hence, allowing the opportunity for unfettered growth. However, by using the shCP gene in the present work, we found that 24 out of 26 clones displaying dark brown color were PCR-positive for the transferred DNA fragment, resulting in less than 10% false-positive transformants. Therefore, we concluded that using this shCP marker gene could greatly reduce the possibility of obtaining false-positive clones in microalgae transgenesis.

### Genomic transformation of transgenic microalgae

According to Bhattacharya et al. [48], the hub of genetic activity occurs in the cell nucleus; thus, nuclear transformation has been the gold standard. On the other hand, Heifetz [49] stated that organelle transformation, which, for example, might involve plant chloroplasts, is characterized by the predictability of transgene expression and the absence of gene silencing, making it particularly useful for increasing plant production or introducing desirable traits. However, the genomic DNA extraction procedure used in our study, as described by Dawson et al. [50], does not distinguish among nucleus, plastids and mitochondrial DNAs. Accordingly, the transformed cells we obtained could represent either nuclear or organellar genome transformation. Thus, the dynamic phenotypes observed in the transformed cells could have resulted from the transformation of the organellar genomes, as represented by colonies that showed mosaic-like patterns with varying color intensities, indicating either gain or loss of transformed gene in consecutive generations. To confirm whether transformed microalgae obtained in our study were the product of nuclear or organellar genome transformation, we would need to further analyze the flanking sequences from the insertion site of the transformed gene.

### shCP is a simple, economical and safe selection marker gene for transgenic microalgae

In this study, the shCP cDNA-encoded purple chromophore protein shCP was used to identify microalgae transformants by simple visual detection of clones that present a dark brown color, eliminating the need for fluorescent microscopy equipment. Moreover, shCP cDNA is a safe selection marker gene since antibiotics, herbicides and drug-resistant chemicals are not needed to distinguish the transformed cells. Instead, the safety of shCP protein encoded by shCP cDNA for commercial use is ensured because it originates from marine anemones, which are edible and biodegradable proteins. Thus, the use of a chromophore as a selection marker appears to work well in haploid cells such as *N*. *oculata*. However, it should further study whether chromophores may work as well in diploid organisms or organelles having multiple copies of the genome.

### Enhanced expression of shCP marker gene in transgenic *N*. *oculata*

It has been reported that optimizing the codon usage of transferred genes before gene transfer can improve foreign protein expression and reduce exogenous gene silencing [51]. Several software programs have been developed to estimate the Codon Adaptation Index (CAI), which is used as a quantitative tool to predict foreign gene expression based on codon usage among different organisms. The CAI value ranges from 0 to 1, where the higher score represents the greater expression of foreign gene, and the lower score represents the reverse. In this study, we isolated the shCP gene from the sea anemone *S*. *haddoni* and transferred it into the microalga *N*. *oculata*. Although both anemone and microalga are eukaryotes, they have their own codon preference. When we used E-CAI (http://genomes.urv.es/CAIcal/E-CAI) software to calculate the CAI value of the shCP gene, it was found to be 0.256. This value is defined as a low translation level, indicating that the shCP gene may not yield efficient expression in microalgae. Nevertheless, as shown in Fig. 2, the expression of exogenous shCP gene in transgenic lines CP5, CP10, CP15, CP19 and CP24 is clear enough to be visible to the naked eye as a dark brown color. Still, we do not rule out the possibility that the expression level of shCP in some colonies might be too low to be recognized as transformed clones. To solve this problem, we can optimize the shCP exogenous gene sequence to adjust for codons frequently used by microalgae. We hypothesize that the use of such modified shCP gene might effectively increase expression intensity, thereby causing enhanced coloration of shCP in transgenic microalgae and, hence, solving the problem described above.

## Materials and Methods

### Plasmid construction

Plasmid pET-15b-shCP, which was first constructed by Chiang et al. [33], served as a template to amplify shCP sequence by using sense primer 5’-GCTAGCCGCCACCATGGCCGGTTTGTTGAAA-3’ (a *Nhe*I cutting site and a 7-bp Kozak sequence are included) and antisense primer 5’-GAATTCTCAATTTGCTTTTTCAGGAAGATCACTGTA-3’ (an *Eco*RI cutting site is included). The PCR reaction was performed under the following conditions: 1 cycle of denaturation at 94°C for 5 min, 25 cycles of denaturation at 94°C for 30 sec and annealing at 58°C for 30 sec and extension at 72°C for 45 sec, followed by 1 cycle of final extension at 72°C for 5 min. The resultant 703-bp shCP PCR product, which was extracted from a 1.5% agarose gel after electrophoresis, was cloned into plasmid pGEM-T easy. The resultant plasmid was digested with *Nhe*I and *Eco*RI to obtain the shCP fragment. The shCP fragment was then inserted into plasmid phr-LFB-DsRed [10]. This expression vector was driven by an algae inducible promoter, *HSP70A* plus *RBCS2*, which was obtained by cutting *Nhe*I and *Eco*RV from pCB740 vector, an artificial promoter from *C*. *reinhardtii* which can improve transgene expression in algae [52]. After the phr-LFB-DsRed vector was digested with *Nhe*I and *EcoR*I, an inducible promoter, HSP70A::RBCS2, and backbone were preserved, but the DsRed fragment was removed. The final expression plasmid, phr-shCP, was generated. This plasmid was linearized by *Sac*II for transformation.

### Culture conditions of *N*. *oculata* NIES-2146

*N*. *oculata* NIES-2146 were obtained from the National Institute for Environmental Studies. The cells were cultured in f/2 medium under continuous light with a photon flux density of 85 μmol photons m^−2^ s^−1^ at 28°C. Agar plates were prepared with 0.8% agar (Sigma) in f/2 medium [53] with seawater and maintained at the same light intensity at 26°C.

### Protoplast preparation

The procedure to produce *N*. *oculata* protoplasts was followed by Li and Tsai [10] with some modification. To prepare protoplasts, *N*. *oculata* cells were grown in f/2 medium at mid-log phase (∼2 × 10^6^ cells/mL). Then 5 × 10^8^ cells were collected and the pellet was washed twice with 1 ml of sterilized seawater, followed by resuspension in 500 μL of synthetic gastric juice (pH 2.0) with 5% pepsin (Sigma) to digest microalgae cell walls [54] and, finally, incubation at 37°C for one hr in dark with gentle shaking. After treatment, cells were washed twice with 1 ml of sterilized seawater to terminate enzymatic activity. The pellet was washed once with 1 ml of electroporation buffer containing 0.08 mM KCl, 0.005 mM CaCl_2_, 0.01 mM HEPES, 0.2 mM Mannitol and 0.2 mM Sorbitol [55] and finally resuspended in 200 μL of electroporation buffer and chilled on ice for 10 min to prepare for electroporation.

### Genetic transformation and selection of the transformed colonies

The transformation of *N*. *oculata* followed procedures previously described by Li and Tsai [10]. Briefly, all *N*. *oculata* protoplasts (5 × 10^8^ cells) in a volume of 200 μL were resuspended in the electroporation buffer. Half of them (2.5 × 10^8^ cells) were taken and added to 10 μg of linearized plasmid phr-shCP which was used for each electroporation within one hr. Electroporation was performed with an electroporator in a 1-mm cuvette (T820, BTX, USA). The electroporator was adjusted to 2,000V voltage, 25 μs pulse length, and 10 pulse time. After electroporation, cells were transferred to a 15 mL glass tube which contained 5 mL f/2 medium and incubated in low light overnight. Then, all cells (2.5 × 10^8^ cells) were plated on an f/2 agar plate for two weeks, followed by counting single algal cells exhibiting green or slightly dark green coloration. Those algal cells that displayed a slightly dark green color were individually selected, given a name and continuously subcultured on a fresh f/2 agar plate for two successive weeks. They were then treated with heat-shock at 42°C for five hrs. After heat-shock treatment, colonies were cultured for one more week. Afterwards, cells that displayed a dark brown color were selected for further confirmation by DNA analysis. These steps were carried out twice under the same conditions.

### Genomic DNA extraction

The extraction of genomic DNA of *N*. *oculata* followed the description of [50] with modification. Briefly, 10 mL of cell culture (∼1 × 10^8^) were harvested and resuspended in 350 μL hexadecyltrimethylammonium bromide buffer by the following protocol. After the mixture was incubated at 65°C for 30 min, the lysate was mixed with 1 volume of phenol/chloroform and left to stand 5 min at room temperature, followed by centrifugation at 13,000 x g at 25°C for 10 min and extraction of aqueous layer to a new Eppendorf. Finally, the genomic DNA was precipitated by 1/10 volume of sodium acetate and 2 volumes of 99.5% ethanol in -20°C for at least 90 min, centrifuged at 13,000 x g at 4°C for 10 min, washed with 70% ethanol to remove salt, and centrifuged again at 13,000 x g at 4°C for 5 min. Finally, the pellet was dried for 3 min to remove additional ethanol and then resuspended in 25 μL of distilled deionized *water*.

### PCR used to detect positive transformants

The existence of plasmid phr-shCP in microalgae was determined by PCR analysis using genomic DNAs as templates to amplify the coding region of shCP cDNA when the forward primer 5’-ATGGCCGGTTTGTTGAAAGAAA-3’ and the reverse primer 5’-TCAATTTGCTTTTTCAGGAAGATCA-3’ were used. Each PCR consisted of 50 μL of solution containing 20 ng of template, 10 μmol of each primer, 25 mM of each dNTP, and 5 units of Taq enzyme in a 10 X PCR buffer (GenTaq, Taiwan). Amplification was performed with 35 cycles of denaturation at 94°C for 1 min, annealing at 62°C for 1 min, and extension at 72°C for 80 sec, followed by 7 min extension at 72°C. The PCR products were subjected to electrophoresis on a 0.8% agarose gel (MDBio, Taiwan).

### Screening the putative transgenic *N*. *oculata* by stereological image microscopy

The putative transgenic clones of microalgae were also observed by a stereological image system to visualize the transgenic clones containing phr-shCP. Images were captured with an Applied Precision Spectris optical sectioning microscope system equipped with a stereo investigator and neurolucida microscopy, an Olympus Plan Apo 1006 oil immersion objective, and Qimaging digital camera. Using stereological image software, the brightness and contrast were adjusted, setting the area outside of cells as background.

### Western blot analysis of transgenic *N*. *oculata*

When transgenic line CP10 was subcultured at the 12^th^ round of replating, we picked up ten single colonies and cultured them separately on the plate. After cultivation for three weeks, algal cells were harvested from these ten separate single colonies, transferred into a 15 mL glass tube containing 5 mL f/2 medium, and continuously cultivated for three weeks without shaking. After that, all algal cells were inoculated into 50 mL of fresh f/2 medium and cultivated for another three weeks. Then, all algal cells were continuously enriched in 100 mL of fresh f/2 medium for one week. Finally, all algal cells were inoculated into 500 mL of fresh f/2 medium for three weeks. Total proteins were extracted from these cultured *N*. *oculata* using the protocol described by Mayfield and Schultz [56] with some modifications. Briefly, the microalgae were collected by centrifuging for 10 min at 8,000 x g at 4°C, and the pellet was resuspended in 100 μL of algal protein extraction buffer (750 mM Tris-HCl (pH 8.0), 15% sucrose (w/v), 100mM β-mercaptoethanol and 1 mM phenylmethylsulfonylfluoride). The cell suspension was transferred to a microtube and broken by glass beads (Sigma, USA) at 4°C for 1 min with a 1 min interval. This process was repeated seven times. Then, the supernatant was centrifuged for 10 min at 13,000 x g at 4°C. The amounts of total soluble proteins were quantified by the Bradford method (Bio-Rad, USA). Samples of 60 μg proteins from wild-type, 50 μg proteins from transgenic line CP10, and 0.1 μg of recombinant His-shCP were individually added into sample loading buffer (1 mM EDTA, 250 mM Tris–HCl (pH 6.8), 4% SDS, 2% β-mercaptoethanol, 0.2% bromophenol blue and 50% glycerol) in a volume one quarter that of the extraction buffer. Samples were boiled for 10 min. The supernatant was electrophoresed on a 12% SDS-PAGE. The proteins were transferred to a polyvinyl difluoride membrane (Millipore) which was blocked with Tris-buffered saline containing 10% skim milk and 0.1% Tween 20 for 1 h at room temperature, followed by immunoblotting with a rabbit polyclonal antibody against shCP (AbKing, Taiwan) with a dilution of 1:10,000 at 4°C overnight. Goat antibody against GAPDH (Santa Cruz, USA) with dilution of 1:2500 served as the internal control. After washing, membranes were incubated with a horseradish peroxidase conjugated goat anti-rabbit (1:5000, Santa Cruz, USA) and goat anti-mouse IgG secondary antibody (1:5000, Santa Cruz, USA) for 1 h, respectively. Bands recognized by antibodies were detected using enhanced chemiluminescent reagents (Advansta) and photographed with FUJIFILM medical X-Ray film. For positive control, the recombinant His-tagged shCP protein was produced by an *E*. *coli* system and purified by His GraviTrap (GE Healthcare).

## Supporting Information

S1 File Genetic Transformation and selection of *N. oculata*. (DOCX)

S1 Fig Selection of the transformed *N. oculata*: the first trial. After electroporation, a total of 2.5 x 10^8^ algal cells were cultured on f/2 medium plate for two weeks. (A) A total of 94 colonies were grown. Most of them exhibited a green coloration. However, 26 colonies displayed a slightly dark green color. (B) These 26 colonies were individually selected, given a name and continuously subcultured on a fresh f/2 medium plate. After two weeks of cultivation, each colony grew normally, and they were ready for further heat-shock treatment.
(DOCX)

S2 Fig Selection of the transformed *N. oculata*: the second trial. After electroporation, a total of 2.5 x 10^8^ algal cells were cultured on f/2 medium plate for two weeks. A total of 384 colonies were grown. Most of them exhibited a green coloration. However, 15 colonies displayed a slightly dark green color. (B) These 15 colonies were individually selected, given a name and continuously subcultured on a fresh f/2 medium plate. After two weeks of cultivation, each colony grew normally, and they were ready for further heat-shock treatment.
(DOCX)

S3 Fig Using purple chromoprotein to screen and isolate the transformed colonies in the 2^nd^ trial. (A) After electroporation with plasmid phr-shCP, 15 colonies of *N. oculata* were grown on f/2 medium plates for two weeks, followed by heat-shock at 42°C for 5 hr. After cultivation for one week, a dark brown coloration presented by reporter chromoprotein was observed in some cells in each colony, as indicated by red arrows. The wild-type colony served as a negative control. (B) The putative transformants of *N. oculata* were picked up and cultivated on the second round of replating (G2), the third round of replating (G3) and so on until the fifteenth round of replating (G15) within 17 months. In contrast to the green coloration presented in the wild-type strain after heat-shock treatment (HS), a dark brown coloration was exhibited in the whole colony of transgenic strains CP2^nd^ 1, CP2^nd^ 4∼5 and CP2^nd^7∼14. “Stable” indicates that foreign protein could be continuously expressed beyond the 15th round of replating.
(DOCX)

S4 Fig PCR detection of the transferred gene in the transformed cells obtained from the 2^nd^ trial. Plasmid phr-shCP was used to transform *N. oculata*. After transformation, all microalgal cells were cultured, and the putative clones were selected to determine the existence of the shCP gene in *N. oculata*. The wild-type strain served as a negative control, whereas plasmid phr-shCP served as a positive control. The expected molecular size of phr-shCP after PCR amplification was 0.684 kb. The small subunit 18S ribosomal RNA (18s) served as the internal control.
(DOCX)

S1 Table Transformation and selection of *N. oculata* harboring shCP marker. (DOCX)

## References

[1] Hare P, Chua NH. Excision of selectable marker genes from transgenic plants. Nat Biotechnol. 2002;20: 575–580. 12042860 10.1038/nbt0602-575

[2] Fernandez E, Schnell R, Ranum LP, Hussey SC, Silflow CD, Lefebvre PA. Isolation and characterization of the nitrate reductase structural gene of Chlamydomonas reinhardtii. Proc Natl Acad Sci USA. 1989; 86: 6449–6453. 2475871 10.1073/pnas.86.17.6449 PMC297861

[3] Debuchy R, Purton S, Rochaix JD. The argininosuccinate lyase gene of Chlamydomonas reinhardtii: an important tool for nuclear transformation and for correlating the genetic and molecular maps of the ARG7 locus. EMBO J. 1989;8: 2803–2809. 2583083 10.1002/j.1460-2075.1989.tb08426.x PMC401327

[4] Nelson JA, Savereide PB, Lefebvre PA. The CRY1 gene in Chlamydomonas reinhardtii: structure and use as a dominant selectable marker for nuclear transformation. Mol Cell Biol. 1994;14: 4011–4019. 8196640 10.1128/mcb.14.6.4011-4019.1994 PMC358767

[5] Stevens DR, Rochaix JD, Purton S. The bacterial phleomycin resistance gene ble as a dominant selectable marker in Chlamydomonas. Mol Gen Genet. 1996;251: 23–30. 8628243 10.1007/BF02174340

[6] Hall LM, Taylor KB, Jones BD. Expression of a foreign gene in Chlamydomonas reinhardtii. Gene. 1993;124: 75–81. 8382657 10.1016/0378-1119(93)90763-s

[7] Sizova I, Fuhrmann M, Hegemann P. A Streptomyces rimosus aphVIII gene coding for a new type phosphotransferase provides stable antibiotic resistance to Chlamydomonas reinhardtii. Gene. 2001;277: 221–229. 11602359 10.1016/s0378-1119(01)00616-3

[8] Miki B, McHugh S. Selectable marker genes in transgenic plants: applications, alternatives and biosafety. J Biotechnol. 2004;107: 193–232. 14736458 10.1016/j.jbiotec.2003.10.011

[9] Franklin S, Ngo B, Efuet E, Mayfield SP. Development of a GFP reporter gene for Chlamydomonas reinhardtii chloroplast. Plant J. 2002;30: 733–744. 12061904 10.1046/j.1365-313x.2002.01319.x

[10] Li SS, Tsai HJ. Transgenic microalgae as a non-antibiotic bactericide producer to defend against bacterial pathogen infection in the fish digestive tract. Fish Shellfish Immunol. 2009;26: 316–325. 10.1016/j.fsi.2008.07.004 18691655

[11] Lauersen KJ, Berger H, Mussgnug JH, Kruse O. Efficient recombinant protein production and secretion from nuclear transgenes in Chlamydomonas reinhardtii. J Biotechnol. 2013;167: 101–110. 10.1016/j.jbiotec.2012.10.010 23099045

[12] Walker TL, Purton S, Becker DK, Collet C. Microalgae as bioreactors. Plant Cell Rep. 2005;24: 629–641. 16136314 10.1007/s00299-005-0004-6

[13] Apt KE, Kroth-Pancic PG, Grossman AR. Stable nuclear transformation of the diatom Phaeodactylum tricornutum. Mol Gen Genet. 1996;252: 572–579. 8914518 10.1007/BF02172403

[14] Allnutt FCT, Kyle DJ, Grossman AR, Apt KE. Methods and tools for transformation of eukaryotic algae. United States of America Patent Number 6027900. 2000.

[15] Srivastava V, Anderson OD, Ow DW. Single-copy transgenic wheat generated through the resolution of complex integration patterns. Proc Natl Acad Sci USA. 1999;96: 11117–11121. 10500139 10.1073/pnas.96.20.11117 PMC17996

[16] Scutt CP, Zubko E, Meyer P. Techniques for the removal of marker genes from transgenic plants. Biochimie, 2002;84: 1119–1126. 12595140 10.1016/s0300-9084(02)00021-4

[17] Day A, Goldschmidt-Clermont M. The chloroplast transformation toolbox: selectable markers and marker removal. Plant Biotechnol. 2011;9: 540–553. 10.1111/j.1467-7652.2011.00604.x 21426476

[18] Fischer N, Stampacchia O, Redding K, Rochaix JD. Selectable marker recycling in the chloroplast. Mol Gen Genet. 1996;251: 373–380. 8676881 10.1007/BF02172529

[19] Rochaix JD Chloroplast reverse genetics: new insights into the function of plastid genes. Trends Plant Sci. 1997;2: 419–425.

[20] Dale EC, Ow DW. Gene transfer with subsequent removal of the selection gene from the host genome. Proc Natl Acad Sci USA. 1991;88: 10558–10562. 1660141 10.1073/pnas.88.23.10558 PMC52968

[21] Landy A. Dynamic, structural, and regulatory aspects of λ site-specific recombination. Ann Rev Biochem. 1989;58: 913–949. 2528323 10.1146/annurev.bi.58.070189.004405

[22] Onouchi H, Nishihama R, Kudo M, Machida Y, Machida C. Visualization of site-specific recombination catalyzed by a recombinase from Zygosaccharomyces rouxii in Arabidopsis thaliana. Mol Gen Genet. 1995;247: 653–660. 7616956 10.1007/BF00290396

[23] Zuo J, Niu QW, Simon GM, Chua NH. Chemical-regulated, site-specific DNA excision in transgenic plants. Nat. Biotechnol. 2001;19: 157–161. 11175731 10.1038/84428

[24] Zhang W, Subbarao S, Addae P, Shen A, Armstrong C, Peschke V, et al Cre/lox mediated marker gene excision in transgenic maize (Zea mays L.) plants. Theor Appl Genet, 2003;107: 1157–1168. 14513214 10.1007/s00122-003-1368-z

[25] Sreekala C, Wu L, Gu K, Wang D, Tian D, Yin Z. Excision of a selectable marker in transgenic rice (Oryza sativa L.) using a chemically regulated Cre/loxP system. Plant Cell Rep. 2005;24: 86–94. 15662501 10.1007/s00299-004-0909-5

[26] Yau YY, Stewart CN Jr. Less is more: strategies to remove marker genes from transgenic plants. BMC Biotechnol. 2013;13: 36 10.1186/1472-6750-13-36 23617583 PMC3689633

[27] Klaus SMJ, Huang FC, Golds TJ, Koop HU. Generation of marker-free plastid transformants using a transiently cointegrated selection gene. Nat Biotechnol. 2004;22: 225–229. 14730316 10.1038/nbt933

[28] Wang HH, Yin WB, Hu ZM. Advances in chloroplast engineering. J Genet Genomics. 2009;36: 387–398. 10.1016/S1673-8527(08)60128-9 19631913

[29] Upadhyaya CP, Nookaraju A, Gururani MA, Upadhyaya C, Kim DH, Chun SC, et al An update on the progress towards the development of marker-free transgenic plants. Bot Stud. 2010;51: 277–292.

[30] Woo HJ, Cho HS, Lim SH, Shin KS, Lee SM, Lee KJ, et al Auto-excision of selectable marker genes from transgenic tobacco via a stress inducible FLP/FRT site-specific recombination system. Transgenic Res. 2009;18: 455–465. 10.1007/s11248-008-9236-x 19160066

[31] Coppoolse ER, de Vroomen MJ, Roelofs D, Smit J, van Gennip F, Hersmus BJ, et al Cre recombinase expression can result in phenotypic aberrations in plants. Plant Mol Biol. 2003;51: 263–279. 12602884 10.1023/a:1021174726070

[32] Kilian O, Benemann CSE, Niyogi KK, Vick B. High-efficiency homologous recombination in the oil-producing alga Nannochloropsis sp. Proc Natl Acad Sci USA. 2011;108: 21265–21269. 10.1073/pnas.1105861108 22123974 PMC3248512

[33] Chiang CC, Chen YL, Tsai HJ. Different visible colors and green fluorescence were obtained from the mutated purple chromoprotein isolated from sea anemone. Mar Biotechnol. 2014;16: 436–446. 10.1007/s10126-014-9563-2 24488042

[34] Shields RJ, Lupatsch I. Algae for aquaculture and animal feeds. J Anim Sci. 2012;4: 23–27.

[35] Sukenik A, Wahnon R. Biochemical quality of marine unicellular algae with special emphasis on lipid composition. I. Isochrysis galbana. Aquaculture. 1991;97: 61–72.

[36] Yamaguchi K. Recent advances in microalgal bioscience in Japan, with special reference to utilization of biomass and metabolites: a review. J App Phycol. 1997;8: 487–502.

[37] Apt KE, Behrens PW. Commercial developments in microalgal biotechnology. J Phycol. 1999;35: 215–226. 10478801

[38] Lavens P, Sorgeloos P. Manual on the production and use of live food for aquaculture. FAO Fisheries Technical Paper No. 361. FAO Rome. 1996.

[39] Trautwein EA. n-3 Fatty acids—Physiological and technical aspects for their use. Eur J Lipid Sci Technol. 2001;103: 45–55.

[40] Duerr EO, Molnar A, Sato V. Cultured microalgae as aquaculture feeds. J Mar Biol. 1998;7: 65–70.

[41] Kropat J, von Gromoff ED, Müller FW, Beck VF. Heat shock and light activation of a Chlamydomonas HSP70 gene are mediated by independent regulatory pathways. Mol Gen Genet. 1995;248: 727–734. 7476876 10.1007/BF02191713

[42] Kropat J, Oster U, Rüdiger W, Beck CF. Chlorophyll precursors are signals of chloroplast origin involved in light induction of nuclear heat-shock genes. Proc Natl Acad Sci USA. 1997;94: 14168–14172. 9391171 10.1073/pnas.94.25.14168 PMC28451

[43] Radakovits R, Jinkerson RE, Fuerstenberg SI, Tae H, Settlage RE, Boore JL, et al Draft genome sequence and genetic transformation of the oleaginous alga Nannochloropis gaditana. Nat Commun. 2012;3: 686 10.1038/ncomms1688 22353717 PMC3293424

[44] Lim FY, Sanchez JF, Wang CC, Keller NP. Toward Awakening Cryptic Secondary Metabolite Gene Clusters in Filamentous Fungi. Methods Enzymol. 2012;517: 303–324. 10.1016/B978-0-12-404634-4.00015-2 23084945 PMC3703436

[45] Coll JM. Methodologies for transferring DNA into eukaryotic microalgae. Span J Agric Res. 2006;4: 316–330.

[46] Mats B, Juan LGP, Guillermo GR, Marianne P. Protoplast isolation from Ulva rigida (Chlorophyta). Br phycol J. 2002;2: 401–407.

[47] Chen HC, Melis A. Marker-free genetic engineering of the chloroplast in the green microalga Chlamydomonas reinhardtii. Plant Biotechnol J. 2013;11: 818–825. 10.1111/pbi.12073 23647698

[48] Bhattacharya A, Kumar A, Desai N, Parikh S. Organelle transformation. Methods Mol Biol. 2012;877: 401–416. 10.1007/978-1-61779-818-4_29 22610643

[49] Heifetz PB. Genetic engineering of the chloroplast. Biochimie. 2000;82: 655–666. 10946114 10.1016/s0300-9084(00)00608-8

[50] Dawson HN, Burlingame R, Cannons AC. Stable transformation of Chlorella: rescue of nitrate reductase-deficient mutants with the nitrate reductase gene. Curr Microbiol. 1997;35: 356–362. 9353220 10.1007/s002849900268

[51] Heitzer M, Eckert A, Fuhrmann M, Griesbeck C. Influence of codon bias on the expression of foreign genes in microalgae. Adv Exp Med Biol. 2007; 616: 46–53. 18161490 10.1007/978-0-387-75532-8_5

[52] Schroda M, Blöcker D, Beck CF. The HSP70A promoter as a tool for the improved expression of transgenes in Chlamydomonas. Plant J. 2000;221: 121–131. 10.1046/j.1365-313x.2000.00652.x 10743653

[53] Guillard RRL. New York Plenum Press. Culture of Phytoplankton for feeding marine invertebrates. In: Smith WL, Chanley HH editors. Culture of Marine Invertebrate Animal. 1975;pp. 29–60.

[54] Atkins TW. Biodegradation of polyethylene adipate microcapsules in physiological media. Biomaterials. 1998;19: 61–67. 9678851 10.1016/s0142-9612(97)00156-7

[55] Chen Y, Wang Y, Sun Y, Zhang Z, Li W. Highly efficient expression of rabbit neutrophil peptide-1 gene in Chlorella ellipsoidea cells. Curr Genet. 2001;39: 365–370. 11525411 10.1007/s002940100205

[56] Mayfield SP, Schultz J. Development of a luciferase reporter gene, luxCt, for Chlamydomonas reinhardtii chloroplast. Plant J. 2004;37: 449–458. 14731263 10.1046/j.1365-313x.2003.01965.x

