# Supplementary material for: Purple Chromoprotein Gene Serves as a New Selection Marker for Transgenesis of the Microalga Nannochloropsis oculata
Source: PLoS One. 2015 Mar 20;10(3):e0120780. doi: 10.1371/journal.pone.0120780 (PMC4368691; doi:10.1371/journal.pone.0120780)
Supplement: S1 File — (DOCX) [file pone.0120780.s001.docx]

**Information S1**

**Genetic Transformation and selection of *N. oculata***

All *N. oculata* protoplasts (5 × 10^8^ cells) in a volume of 200 μL were resuspended in the electroporation buffer. Half of them (2.5 × 10^8^ cells) were taken and added to 10 μg of linearized plasmid phr-shCP which was used for each electroporation within one hr. Electroporation was performed with an electroporator in a 1-mm cuvette (T820, BTX, USA). The electroporator was adjusted to 2,000V field strength, 25 μs pulse length, and 10 pulse time. After electroporation, cells were transferred to a 15 mL glass tube which contained 5 mL f/2 medium and incubated in low light overnight. Then, all cells (2.5 × 10^8^ cells) were plated on an f/2 agar plate for two weeks, followed by individually counting the algal cells exhibiting green, or slightly dark green, coloration. Algal cells that displayed a slightly dark green were individually selected, given a name and continuously subcultured on a fresh f/2 agar plate for two successive weeks. Then, they were treated with heat-shock at 42℃ for five hrs. After heat-shock treatment, colonies were cultured for one more week. Afterwards, cells that displayed a dark brown color were selected for further confirmation by DNA analysis. These steps were carried out twice under the same conditions.
