# Supplementary figures and images for: Purple Chromoprotein Gene Serves as a New Selection Marker for Transgenesis of the Microalga Nannochloropsis oculata
Source: PLoS One. 2015 Mar 20;10(3):e0120780. doi: 10.1371/journal.pone.0120780 (PMC4368691; doi:10.1371/journal.pone.0120780)

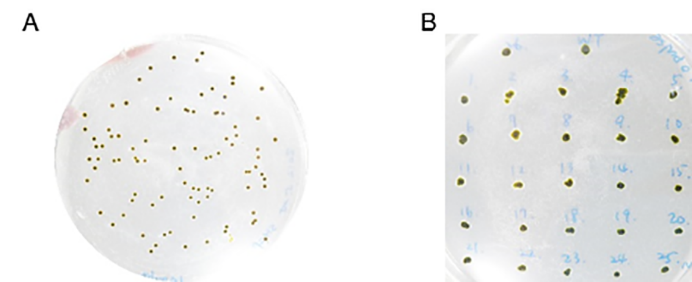


**Figure S1. Selection of the transformed *N. oculata*: the first trial.**

Supplement: S1 Fig — After electroporation, a total of 2.5 x 10^8^ algal cells were cultured on f/2 medium plate for two weeks. (A) A total of 94 colonies were grown. Most of them exhibited a green coloration. However, 26 colonies displayed a slightly dark green color. (B) These 26 colonies were individually selected, given a name and continuously subcultured on a fresh f/2 medium plate. After two weeks of cultivation, each colony grew normally, and they were ready for further heat-shock treatment. (DOCX) [file pone.0120780.s002.docx]

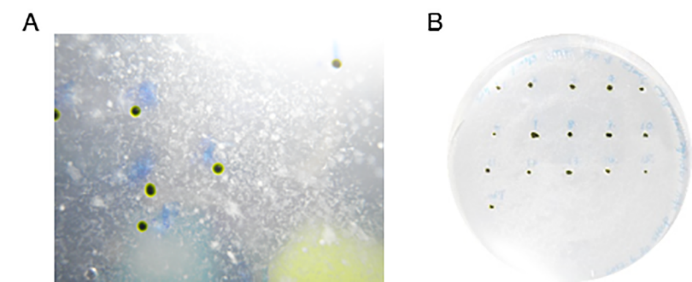


**Figure S2. Selection of the transformed *N. oculata*: the second trial.**

Supplement: S2 Fig — After electroporation, a total of 2.5 x 10^8^ algal cells were cultured on f/2 medium plate for two weeks. A total of 384 colonies were grown. Most of them exhibited a green coloration. However, 15 colonies displayed a slightly dark green color. (B) These 15 colonies were individually selected, given a name and continuously subcultured on a fresh f/2 medium plate. After two weeks of cultivation, each colony grew normally, and they were ready for further heat-shock treatment. (DOCX) [file pone.0120780.s003.docx]
