# Supplementary material for: Purple Chromoprotein Gene Serves as a New Selection Marker for Transgenesis of the Microalga Nannochloropsis oculata
Source: PLoS One. 2015 Mar 20;10(3):e0120780. doi: 10.1371/journal.pone.0120780 (PMC4368691; doi:10.1371/journal.pone.0120780)
Supplement: S3 Fig — (A) After electroporation with plasmid phr-shCP, 15 colonies of N. oculata were grown on f/2 medium plates for two weeks, followed by heat-shock at 42°C for 5 hr. After cultivation for one week, a dark brown coloration presented by reporter chromoprotein was observed in some cells in each colony, as indicated by red arrows. The wild-type colony served as a negative control. (B) The putative transformants of N. oculata were picked up and cultivated on the second round of replating (G2), the third round of replating (G3) and so on until the fifteenth round of replating (G15) within 17 months. In contrast to the green coloration presented in the wild-type strain after heat-shock treatment (HS), a dark brown coloration was exhibited in the whole colony of transgenic strains CP2nd 1, CP2nd 4∼5 and CP2nd7∼14. “Stable” indicates that foreign protein could be continuously expressed beyond the 15th round of replating. (DOCX) [file pone.0120780.s004.docx]

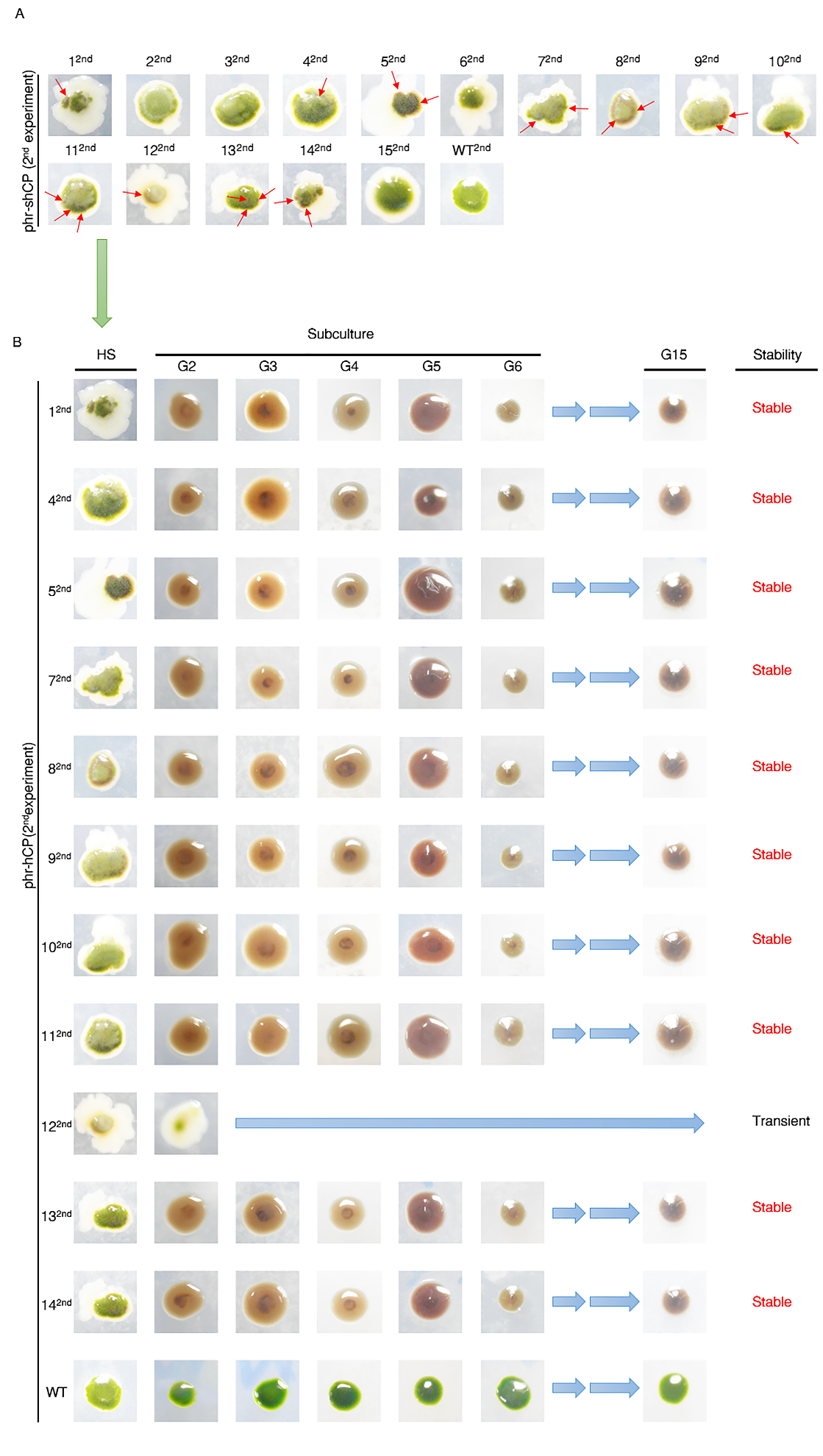


**Figure S3. Using purple chromoprotein to screen the transformants of *N. oculata* and isolate the pure colonies containing the exogenous shCP gene in the 2^nd^ trial.**
