# Supplementary material for: Purple Chromoprotein Gene Serves as a New Selection Marker for Transgenesis of the Microalga Nannochloropsis oculata
Source: PLoS One. 2015 Mar 20;10(3):e0120780. doi: 10.1371/journal.pone.0120780 (PMC4368691; doi:10.1371/journal.pone.0120780)
Supplement: S4 Fig — Plasmid phr-shCP was used to transform N. oculata. After transformation, all microalgal cells were cultured, and the putative clones were selected to determine the existence of the shCP gene in N. oculata. The wild-type strain served as a negative control, whereas plasmid phr-shCP served as a positive control. The expected molecular size of phr-shCP after PCR amplification was 0.684 kb. The small subunit 18S ribosomal RNA (18s) served as the internal control. (DOCX) [file pone.0120780.s005.docx]

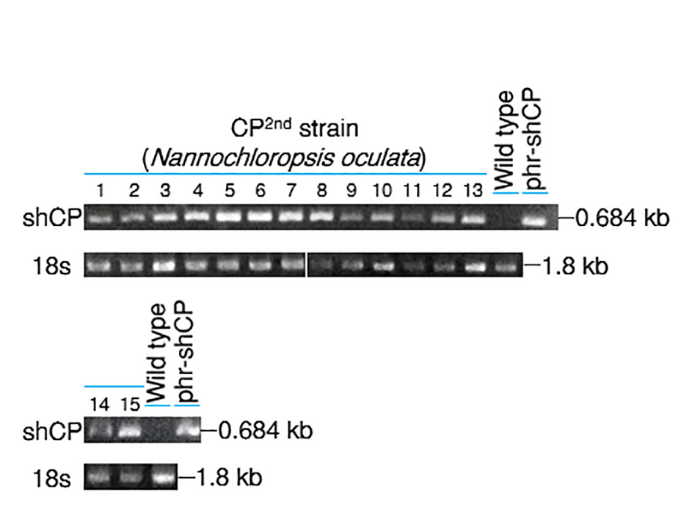


**Figure S4. PCR detection of the transferred gene in the genomic DNAs of the 15 transformed *N. oculata* obtained from the 2^nd^ trial.**
