# Supplementary material for: Purple Chromoprotein Gene Serves as a New Selection Marker for Transgenesis of the Microalga Nannochloropsis oculata
Source: PLoS One. 2015 Mar 20;10(3):e0120780. doi: 10.1371/journal.pone.0120780 (PMC4368691; doi:10.1371/journal.pone.0120780)
Supplement: S1 Table — (DOCX) [file pone.0120780.s006.docx]

**Table S1. Transformation and selection of *N. oculata* harboring shCP marker**

|  | Independent transformation trials^a^ | | |
| --- | --- | --- | --- |
|  | | 1^b^ | 2^c^ |
| Total number of algal cells for electroporation | | 2.5 x 10^8^ | 2.5 x 10^8^ |
| Total number of colonies grown on plate | | 94 | 384 |
| Total number of colonies with dark green coloration^d^ | | 26 | 15 |
| Total number of PCR-positive colonies | | 24 | 15 |
| Total number of colonies with a dark brown coloration^d^ after heat-shock treatment | | 5 | 10 |
| Total number of stable lines | | 2 | 10 |

^a^ Two independent trials of genetic transformation of *N. oculata* with a linearized plasmid phr-shCP.

^b^ The 1^st^ transformation experiment; ^c^ The 2^nd^ transformation experiment.

^d^ By the naked eye.
